# Comprehensive genomic profiling of pulmonary spindle cell carcinoma using tissue and plasma samples: insights from a real‐world cohort analysis

**DOI:** 10.1002/2056-4538.12375

**Published:** 2024-04-25

**Authors:** Yi Sun, Shilei Qin, Song Wang, Jiaohui Pang, Qiuxiang Ou, Weiquan Liang, Hai Zhong

**Affiliations:** ^1^ Department of Pathology The Second Xiangya Hospital of Central South University Changsha PR China; ^2^ Department of Thoracic Surgery Affiliated Hospital of Guilin Medical University Guilin PR China; ^3^ Geneseeq Research Institute Nanjing Geneseeq Technology Inc. Nanjing PR China; ^4^ Department of Respiratory and Critical Care Medicine The Second People's Hospital of Foshan Foshan PR China; ^5^ Department of Thoracic Surgery, Zhujiang Hospital Southern Medical University Guangzhou PR China

**Keywords:** pulmonary spindle cell carcinoma, tumor mutation burden, mutational signature, targeted therapy, chemoimmunotherapy

## Abstract

Pulmonary spindle cell carcinoma (PSCC) is a rare and aggressive non‐small cell lung cancer (NSCLC) subtype with a dismal prognosis. The molecular characteristics of PSCC are largely unknown due to its rarity, which limits the diagnosis and treatment of this historically poorly characterized malignancy. We present comprehensive genomic profiling results of baseline tumor samples from 22 patients histologically diagnosed with PSCC, representing the largest cohort to date. Somatic genetic variant detection was compared between paired plasma samples and primary tumors from 13 patients within our cohort. The associations among genomic features, treatment, and prognosis were also analyzed in representative patient cases. *TP53* (54.5%), *TERT* (36.4%), *CDKN2A* (27.3%), and *MET* (22.7%) were most frequently mutated. Notably, 81.8% of patients had actionable targets in their baseline tumors, including *MET* (22.7%), *ERBB2* (13.6%), *EGFR* (9.1%), *KRAS* (9.1%), *ALK* (9.1%), and *ROS1* (4.5%). The median tumor mutation burden (TMB) for PSCC tumors was 5.5 mutations per megabase (muts/Mb). TMB‐high tumors (>10 muts/Mb) exhibited a significantly higher mutation frequency in genes such as *KRAS*, *ARID2*, *FOXL2*, and *LRP1B*, as well as within the DNA mismatch repair pathway. The detection rates for single nucleotide variants and structural variants were comparable between matched tumor and plasma samples, with 48.6% of genetic variants being mutually identified in both sample types. Additionally, a patient with a high mutation load and positive PD‐L1 expression demonstrated a 7‐month survival benefit from chemoimmunotherapy. Furthermore, a patient with an *ALK*‐rearranged tumor achieved a remarkable 3‐year progression‐free survival following crizotinib treatment. Overall, our findings deepen the understanding of the complex genomic landscape of PSCC, revealing actionable targets amenable to tailored treatment of this poorly characterized malignancy.

## Introduction

Pulmonary sarcomatoid carcinoma (PSC) constitutes less than 1% of all lung cancers; however, it is characterized by highly aggressive clinical behavior with a dismal prognosis [1]. By definition, PSC is a group of poorly differentiated non‐small cell lung cancers (NSCLCs) encompassing five different histological subtypes, namely pleomorphic carcinoma, spindle cell carcinoma, giant cell carcinoma, carcinosarcoma, and blastoma [2]. Pleomorphic carcinoma is the most common type of PSC (>50%), defined as a poorly differentiated NSCLC (adenocarcinoma, squamous cell carcinoma, or large cell carcinoma) containing at least 10% of spindle cell and/or giant cell component, or a carcinoma consisting exclusively of spindle and giant cells [2, 3, 4]. On the other hand, spindle cell carcinoma and giant cell carcinoma are entirely composed of malignant spindle‐shaped and giant cells, respectively, without differentiated carcinomatous elements.

The diagnosis of PSC presents significant challenges, primarily due to its rare incidence, heterogeneous histology, and unclear histogenesis. Most entities under the definition of PSC can be recognized by evaluating morphological features assessed through hematoxylin and eosin (H&E)‐stained slides using a light microscope [5, 6]. In addition, immunohistochemistry (IHC) has become a widely employed ancillary technique to characterize these neoplasms, particularly in cases of small biopsies [4, 7]. The prognosis of PSC patients is generally less favorable compared to stage‐matched patients diagnosed with conventional NSCLC [8, 9]. To date, surgery remains the mainstay in the management of patients diagnosed with PSC, although there have been sporadic reports where patients were treated with chemotherapy, radiotherapy, targeted therapy, and even traditional Chinese medicine [8, 10, 11, 12, 13]. No consensus has been reached on the optimal treatment for PSC patients, primarily due to the rarity of the disease and the lack of comprehensive genetic studies to identify actionable targets. In recent years, promising clinical results have been revealed for immunotherapy, particularly immune checkpoint inhibitors (ICIs), in the treatment of various human cancers [14]. Of interest, Tsurumi *et al* reported a rare case of a patient with pulmonary spindle cell carcinoma (PSCC) who exhibited a remarkable clinical response to pembrolizumab, an ICI targeting programmed cell death‐1 (PD‐1) [15]. Nevertheless, due to the extreme rarity of PSCC, which constitutes approximately 0.1–0.3% of all lung tumors [16, 17], investigations into the genetic events and pathways contributing to its development and progression have been limited. The lack of precision‐based targeted therapy options necessitates more comprehensive genomic profiling to characterize PSCC and identify potential therapeutic targets, thus advancing the management of this poorly studied malignancy.

In this study, we conducted a retrospective analysis of sequencing data derived from baseline tumors within a cohort of 22 patients with histologically confirmed PSCC. This study stands as the largest PSCC patient cohort examined to date, providing a comprehensive genomic characterization of PSCC, which may offer valuable insights into identifying therapeutic targets and developing effective therapeutic strategies for this poorly characterized malignancy.

## Material and methods

### Patients and sample collection

**Figure 1:**
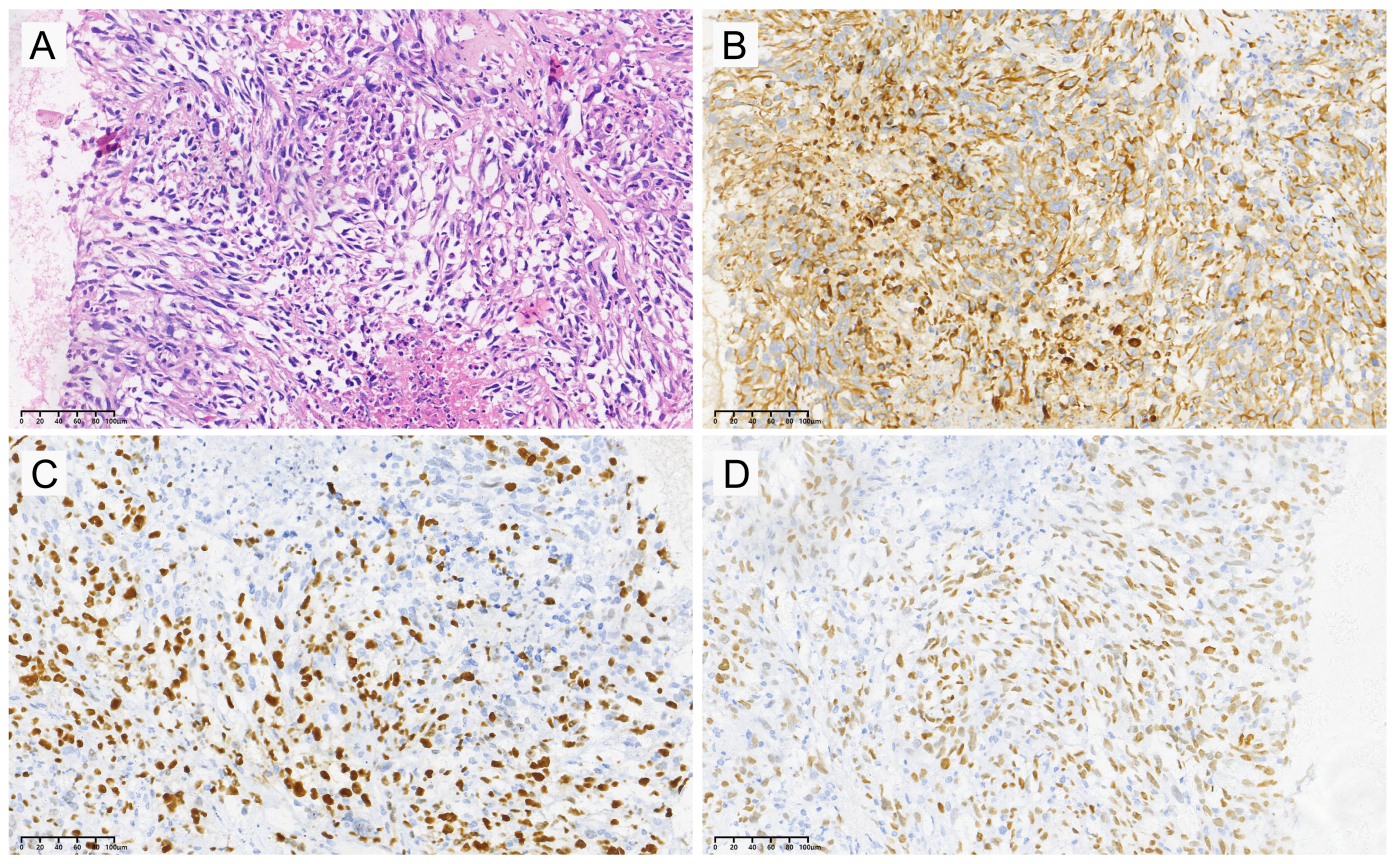
Representative morphological and immunohistochemistry staining images for a patient diagnosed with PSCC. (A) Histopathological image of PSCC. (B–D) Positive immunostaining for cytokeratin 7 (B), Ki‐67 (C), and thyroid transcription factor‐1 (D) reveals the epithelial adenocarcinomatous differentiation of the spindle cell component.

We conducted an extensive database search and identified 22 PSCC patients who were admitted to the participating hospitals between March 2015 and November 2022. Only patients with clinical records indicating a histological diagnosis of PSCC without heterologous sarcomatous elements (such as giant cell carcinoma, pleomorphic carcinoma, carcinosarcoma, or pulmonary blastoma) were included in this study. Tumor and/or plasma samples of these patients were analyzed through targeted next‐generation sequencing (NGS) of 425 cancer‐related genes using the GeneseeqPrime™ panel (Nanjing Geneseeq Technology Inc., Nanjing, PR China) [16]. The diagnosis of PSCC was confirmed by two experienced pathologists (YS and SQ) in a blind fashion. Morphologic features were meticulously assessed on H&E‐stained slides using light microscopy. Immunohistochemical staining for cytokeratin 7, Ki‐67, and thyroid transcription factor‐1 was employed as an ancillary technique to assist in the characterization of various cell components present in these tumors. All tumor samples from the 22 PSCC patients in our study cohort showed positive staining for these three IHC markers (Figure 1). The clinical and genomic data of the external cohort, including 44 PC and 9 PSCC patients, were publicly available [18]. This study was approved by the Medical Ethics Committee of Nanjing Geneseeq Medical Laboratory (NSJB‐MEC‐2023‐04). Written consent form was collected from each patient before sample collection.

### Library preparation and sequencing

DNA extraction, library construction, and sequencing were conducted in a clinical testing laboratory (Nanjing Geneseeq Technology Inc.) accredited by the Clinical Laboratory Improvement Amendments and the College of American Pathologists [19, 20]. Formalin‐fixed paraffin‐embedded (FFPE) samples were de‐paraffinized with xylene, and genomic DNA was extracted using QIAamp DNA FFPE Tissue Kit (Qiagen, Hilden, Germany, Cat. No. 56404), following the manufacturer's instructions. Peripheral blood samples were centrifuged at 1,800 × *g* for 10 min, followed by extraction and purification of cell‐free DNA (cfDNA) using the QIAamp Circulating Nucleic Acid Kit (Qiagen, Cat. No. 55114). Genomic DNA of white blood cells in sediments was used as the normal control, and its extraction was carried out using the DNeasy Blood and Tissue Kit (Qiagen, Cat. No. 69504). Nanodrop2000 (Thermo Fisher Scientific, Waltham, MA, USA) was used to assess the quality of genomic DNA, while the fragment distribution of DNA was analyzed using the High Sensitivity DNA Kit (Agilent Technologies, Santa Clara, CA, USA, 5067‐4626) on a Bioanalyzer 2100. The dsDNA HS assay kit was used to quantify DNA on a Qubit 3.0 fluorometer (Life Technology, Waltham, MA, USA). NGS libraries were constructed using the KAPA Hyper Prep kit (KAPA Biosystems, Wilmington, MA, USA) with an optimized manufacturer's protocol for different sample types. Hybridization‐based target enrichment was carried out with GeneseeqPrime™‐targeted NGS panel and xGen Lock‐down Hybridization and Wash Reagents Kit (Integrated DNA Technologies, Coralville, IA, USA) [21]. The target‐enriched library was then sequenced on the Illumina Hiseq4000 platform according to the manufacturer's instructions.

### Data processing and mutation calling

Sequencing data were demultiplexed and subjected to FASTQ file quality control (QC) using Trimmomatic [22]. Qualified data with a QC score above 15 and did not have extra N bases were mapped to human genome Hg19 using Burrows‐Wheeler Aligner (BWA‐mem, v0.7.12; https://github.com/lh3/bwa/tree/master/bwakit). The base quality score was recalibrated, and local realignment was performed around the insertions/deletions (indels) using the Genome Analysis Toolkit (GATK 3.4.0; https://software.broadinstitute.org/gatk/). Duplicates were removed using Picard. VarScan2 was used to identify single‐nucleotide variations (SNVs) and indel mutations. SNVs with a variant allele frequency of less than 1% were filtered out, and common SNVs present in more than 1% of the population in the 1000 Genomes Project or the Exome Aggregation Consortium 65,000 exomes database were excluded. An in‐house list of recurrent artifacts based on a normal pool of whole blood samples was used to filter the mutation list. Matched white blood cells from each patient were sequenced in parallel to remove sequencing artifacts, germline variants, and clonal hematopoiesis. Copy number variant (CNV) analysis was conducted by adjusting copy number values according to sample ploidy using FACETS [23, 24]. Structural variants (SVs) were detected by FACTERA using default parameters [25]. Fusion reads were manually reviewed and confirmed on Integrative Genomics Viewer [26].

The cutoffs of variant allele frequency (VAF) and sequencing reads/depths for tumor samples were as follows: altered reads ≥3, VAF ≥1%, depth ≥30× for somatic SNVs and indels; split‐reads ≥3 for SVs; CNVs were hot‐spots in the TCGA database [27] with gene ratio ≥2 as copy number gain and gene ratio ≤0.6 as copy number loss. The cutoffs for tumor‐informed matched variants identified in paired plasma samples were as follows: altered reads ≥3, depth ≥30× for somatic SNVs and indels; split‐reads ≥3 for SVs; gene ratio ≥1.6 as copy number gain and gene ratio ≤0.6 as copy number loss. The cutoffs for unique variants identified in plasma samples were as follows: altered reads ≥3, VAF ≥1%, depth ≥30× or altered reads ≥10, depth ≥30× for somatic SNVs and indels; split‐reads ≥3 for SVs; gene ratio ≥1.6 as copy number gain and gene ratio ≤0.6 as copy number loss.

Tumor mutation burden (TMB) was calculated as the total number of nonsynonymous mutations divided by the length of the genomic target region. TMB‐high (TMB‐H) tumors were defined as tumors with TMB > 10 muts/Mb, whereas TMB‐low (TMB‐L) tumors were defined as tumors with TMB ≤ 10 muts/Mb. The microsatellite (MS) status of tumor samples was determined by the overall stability of MS loci covered by the sequencing panel as previously described [28]. In brief, a sample was reported as microsatellite instable (MSI) if the proportion of unstable MS loci was above 40% or as microsatellite stable (MSS) if the proportion of unstable MS loci was below 40%.

### Mutational signature analysis

Tumor samples with a count of ≥5 synonymous/nonsynonymous mutations were included in the mutation signature analysis using the ‘maftools’ and ‘sigminer’ packages in R (version 4.1.3) [29, 30]. The 30 mutational signatures (v2) listed on the COSMIC website (https://cancer.sanger.ac.uk/signatures/signatures_v2/) were classified into 10 groups, including age (COSMIC_1), APOBEC (COSMIC_2 and COSMIC_13), BRCA (COSMIC_3), smoking (COSMIC_4), DNA mismatch repair (MMR) deficiency (COSMIC_6, COSMIC_15, COSMIC_20, and COSMIC_26), ultraviolet (COSMIC_7), POLE (COSMIC_10), temozolomide (COSMIC_11), immunoglobulin (COSMIC_9), and others (the rest of the signatures). The contribution of each mutational signature in each patient was calculated as the proportion of the selected signature over all signatures as previously described [24, 31]. In addition, we also extracted mutational signature features that had the most cosine similarity to the previously defined COSMIC signatures using the ‘NFM’ package in R [32]. The optimal number of signatures was determined by measuring the cophenetic correlation coefficient (range: 0–1), which indicates the robustness of consensus matrix clustering [29].

### RNA sequencing

Total RNA was extracted from FFPE samples using the RNeasy FFPE Kit (Qiagen, Cat. No. 73504) according to the manufacturer's instructions. RNA quality was assessed on an Agilent 2100 Bioanalyzer. Ribosomal RNA and genomic DNA were removed using the KAPA Stranded RNA‐Seq Kit (KAPA Biosystems) with RiboErase (HMR) and DNase digestion, followed by purification using Agencourt RNA Clean XP Beads (Beckman Coulter, Beverly, MA, USA) as per the manufacturer's protocol. Sequencing libraries were constructed using the KAPA Stranded RNA‐Seq Library Preparation Kit (KAPA Biosystems). Targeted hybridization‐based capture of 201 cancer‐related gene transcripts (supplementary material, Table S1) was performed using the Sarcorna™ panel according to the manufacturer's instructions (Nanjing Geneseeq Technology Inc.).

Libraries were sequenced on the Illumina HiSeq NGS platform. Base calling was carried out using bcl2fastq v2.16.0.10 (Illumina, Inc., San Diego, CA, USA) to generate sequencing reads in the FASTQ format. Quality control was performed using Trimmomatic (version 0.33) [22]. Qualified reads were then aligned to the human genome Hg19 using STAR (version 2.5.3a) [33] to identify individual exon, intron, and intergenic features. The average coverage of mapped reads across the base positions of the feature coordinates was subsequently computed. Gene fusions were identified and visualized using the Integrative Genomics Viewer [34].

### Immunohistochemical analysis of PD‐L1 expression

PD‐L1 staining was performed using Dako PD‐L1 IHC 22C3 pharmDx Kit (Agilent Technologies) according to the manufacturer's instructions [35, 36]. The specimen was then counterstained with hematoxylin and coverslipped. The PD‐L1 expression level was determined using the tumor proportion score (TPS), calculated as the number of PD‐L1‐staining positive tumor cells divided by the total number of viable tumor cells multiplied by 100. The value of TPS ≥ 1% was defined as PD‐L1‐positivity. The strong staining observed in the reticulated epithelium of the deep tonsillar crypts was used as the positive control, contrasting with the absence of staining on the surface epithelium of tonsils, which served as the negative control.

### Statistical analysis

All statistical analyses were performed in R (version 4.1.3). Fisher's exact test was used to compare the frequencies of categorical variables among different groups, and the Wilcoxon rank sum test was used to compare the distribution of continuous data. Kaplan–Meier survival curves were used to compare the survival of patient subgroups, and the statistical difference was assessed using the log‐rank test. Hazard ratios with 95% confidence intervals (CIs) were estimated by the Cox proportional hazards model. A two‐sided *p* value of less than 0.05 was considered significant for all tests unless indicated otherwise (*p < 0.05; **p < 0.01; ***p < 0.001).

## Results

### Patient characteristics

Twenty‐two PSCC patients were identified from the extensive search (Figure 1 and supplementary material, Figure S1). There were more males than females (63.6% versus 36.4%), and the median age of diagnosis was 67 years (range: 29–84) (Table 1). Approximately 72.7% of patients had no family history of cancer. All 22 patients were classified as having MSS tumors, with 18 of them exhibiting tumors with a low TMB of ≤10 muts/Mb. The PD‐L1 expression status was available for eight patients within our study cohort, among whom two exhibited a TPS of <1%, three had TPS ranging between 1% and 50%, and three had TPS ≥50%. No significant correlation was found between TMB and PD‐L1 expression levels among PSCC patients (Spearman's rank correlation: rho = 0.26, *p* = 0.54) (supplementary material, Figure S2A). Regarding treatment modalities, three patients received targeted therapy and seven underwent combination therapy, including various combinations of surgery, radiotherapy, chemotherapy, and immunotherapy.

**Table 1:** Clinical characteristics of PSCC patients (*N* = 22)

Characteristic	*N* (%)
Age at diagnosis (years)	
Median (range)	67 (29–84)
≤67	13 (59.1)
>67	9 (40.9)
Sex	
Male	14 (63.6)
Female	8 (36.4)
Clinical stage at diagnosis	
I–III	3 (13.6)
IV	6 (27.3)
Unknown	13 (59.1)
Primary tumor location	
Left lung	9 (40.9)
Right lung	13 (59.1)
Pleural involvement	
Yes	5 (22.7)
No	2 (9.1)
Unknown	15 (68.2)
Family history of cancer	
Yes	6 (27.3)
No	16 (72.7)
MSI status	
MSS	22 (100)
TMB	
Median (range)	5.5 (1.0–20.8)
TMB‐L (≤10 muts/Mb)	18 (81.8)
TMB‐H (>10 muts/Mb)	4 (18.2)
TPS	
TPS < 1%	2 (9.1)
1 ≤ TPS < 50%	3 (13.6)
≥50%	3 (13.6)
Unknown	14 (63.6)
Type of treatment	
Chemoimmunotherapy	1 (4.6)
Radiotherapy	1 (4.6)
Chemotherapy	1 (4.6)
Targeted therapy	3 (13.6)
Combination therapy*	4 (18.2)
Unknown	12 (54.5)

MSI, microsatellite instability; MSS, microsatellite stable; TMB, tumor mutation burden; TPS, tumor proportion score.

* Combination therapy in this study refers to any combination of the following treatment modalities: surgery, radiotherapy, and chemotherapy.

### Unveiling potential therapeutic targets via genetic profiling of PSCC tumors

**Figure 2:**
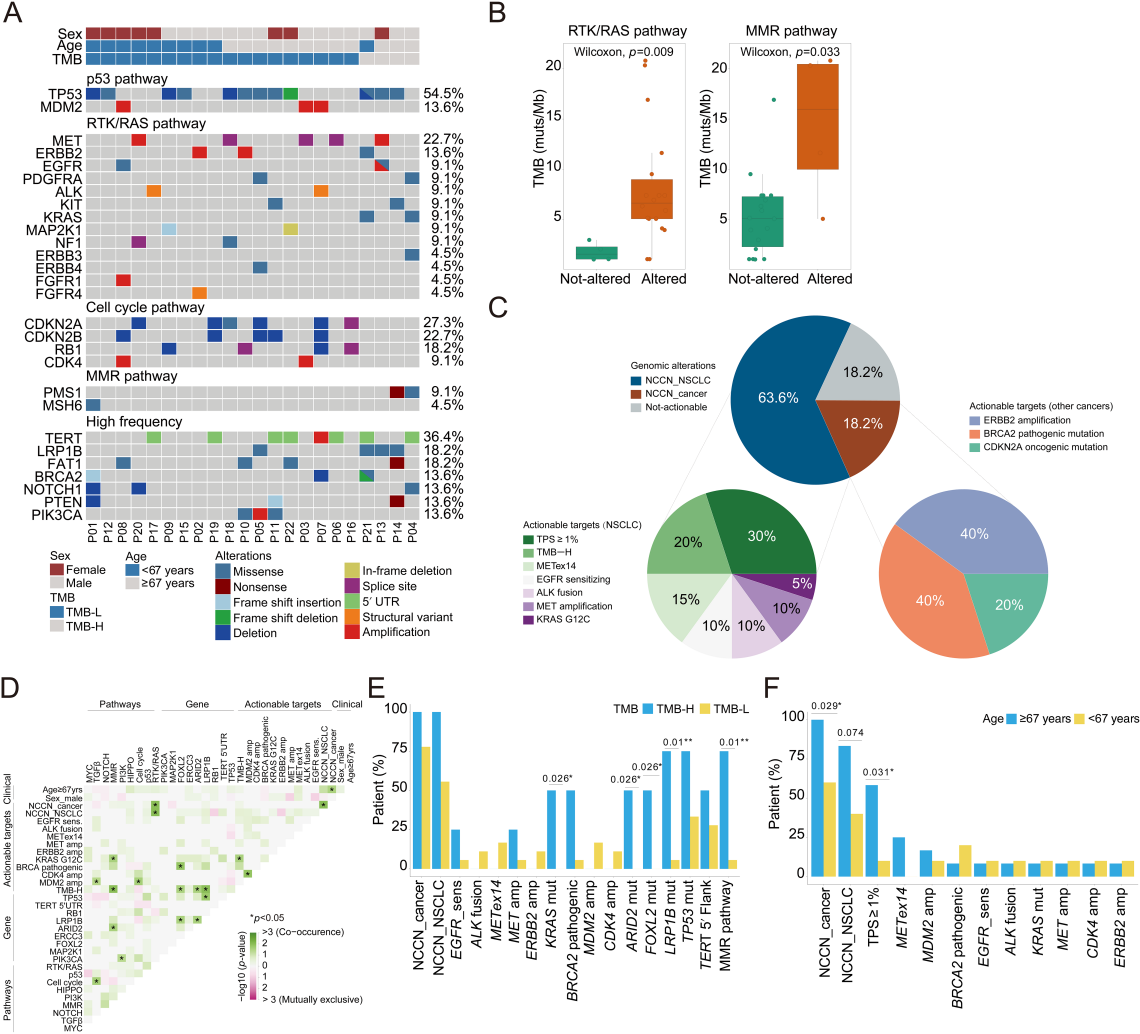
Genomic landscape of PSCC patients. (A) Co‐mutation plot of genetic variants identified from tumor tissues of 22 treatment‐naïve PSCC patients. Each column represents one patient. The mutation frequency of selected genomic alterations is listed to the right of the plot. (B) Box plots show the distribution of TMB in PSCC patients with or without genomic abnormalities in the RTK/RAS or DNA MMR pathway. (C) Pie charts demonstrate the number and percentage of actionable genomic alterations categorized by National Comprehensive Cancer Network guidelines. (D) Co‐occurrence and mutual exclusivity of clinical/molecular features in baseline PSCC tumors. (E) The bar plot shows the proportion of patients carrying the selective genomic features between TMB‐H (>10 muts/Mb) and TMB‐L (≤10 muts/Mb) patients. (F) The bar plot shows the proportion of patients with selected genomic features in subgroups of patients stratified by age.

Within the study cohort, *TP53* (54.5%), *TERT* (36.4%), *MET* (22.7%), *CDKN2A* (27.3%), *CDKN2B* (22.7%), *RB1* (18.2%), *LRP1B* (18.2%), and *FAT1* (18.2%) were among the most frequently mutated genes (Figure 2A). Known oncogenic drivers, such as *EGFR* L858R, *EGFR* amplification, *KRAS* G12C, and *MET* exon14 skipping alterations (*METex14*) were also found in baseline PSCC tumors. Additionally, we report three anaplastic lymphoma kinase (*ALK*) rearrangements in two patients (P07 and P17) through DNA‐based large panel sequencing (Figure 2A). Among these fusion events, two involved partner breakpoints within the intergenic region (IGR), while one occurred within exon 10 of the *TPM3* gene (Table 2). The VAFs of these *ALK* fusions ranged from 14.3% to 66.0%. Since RNA sequencing (RNA‐seq) serves as a powerful tool for identifying and validating gene rearrangements, we then compared the SV detection results between NGS‐based DNA sequencing and RNA‐seq methodologies. It is of note that RNA‐seq was performed using the remaining tumor samples from patients. None of the other patients had sufficient tumor tissues available for further analysis. Remarkably, an *EML4*: exon 13–*ALK*: exon 20 fusion was identified in the tumor sample from P07 at the RNA level (Table 2). EML4, specifically echinoderm microtubule‐associated protein‐like 4, is the first identified and the most common fusion partner of *ALK*, making it a promising actionable target for treatment and a diagnostic biomarker in NSCLC [37]. On the other hand, the tumor sample from P17 yielded negative detection of fusions via RNA‐seq, which we hypothesized could potentially result from suboptimal sample quality for sequencing. Nonetheless, these findings underscore the significance of RNA‐based analysis in detecting SVs in tumor samples.

**Table 2:** Comparison of structural variant detection using DNA sequencing and RNA sequencing methodologies

	DNA sequencing			RNA sequencing	
ID	*ALK* fusion partner	Fusion site	VAF	*ALK* fusion partner	Fusion site
P07	IGR	IGR–*ALK* (downstream *PPP3CA*: exon 20)	66.0%	*EML4*	*EML4*–*ALK* (exon 13: exon 20)
P17	*TPM3*	*TPM3*–*ALK* (exon 10: exon 20)	14.3%	–	–
	IGR	IGR–*ALK* (upstream *TRIB2*: exon 20)	22.8%	–	–

ALK, anaplastic lymphoma kinase; EML4, echinoderm microtubule‐associated protein‐like 4; IGR, intergenic region; PPP3CA, protein phosphatase 3 catalytic subunit alpha; TPM3, tropomyosin 3; TRIB2, tribbles pseudokinase 2; VAF, variant allele frequency.

The median TMB of PSCC tumors in our study cohort was 5.5 muts/Mb. Notably, patients with an altered RTK/RAS or DNA MMR pathway showed a significantly higher TMB compared to those without the altered pathway (*p* = 0.009 and 0.033, respectively) (Figure 2B). Neither sex (*p* = 0.68) nor age (*p* = 0.16) was significantly correlated with TMB in these patients (supplementary material, Figure S2B).

To explore the clinical implications of these mutation events, we categorized genomic alterations based on the level of evidence that a particular molecular feature is recommended for testing in the National Comprehensive Cancer Network (NCCN) guideline [38, 39]. As shown in Figure 2C, 63.6% (14/22) of PSCC patients harbored actionable targets recommended for NSCLC (NCCN_NSCLC), and 18.2% (4/22) had genomic features listed in the NCCN guideline recommended for testing in other cancers (NCCN_cancer). Among all the NCCN_NSCLC targets, 30% were PD‐L1 positive (TPS ≥ 1%; 6/20), followed by TMB‐H (20%, 4/20) and *METex14* (15%, 3/20). Meanwhile, NCCN_cancer targets were mainly composed of *ERBB2* amplification (40%), *BRCA2* pathogenic mutations (40%), and *CDKN2A* oncogenic mutations (20%). Interestingly, we showed that TMB‐H, a biomarker potentially indicative of longer survival and higher response rates with ICI therapy, was more likely to co‐occur with alterations in *FOXL2*, *ARID2*, *RB1*, *KRAS* G12C, and the DNA MMR signaling pathway (Figure 2D,E). Additionally, cell cycle pathway variants were likely to co‐exist with TGFβ pathway gene alterations, suggesting that these two pathways may both contribute to the development and progression of PSCC. Besides, we have observed that drug‐associated genetic variants of cancer and positive PD‐L1 expression were more frequently identified in patients aged 67 years and older (Figure 2F). Conversely, *BRCA2* pathogenic mutation seemed to be more prevalent in younger patients, although the difference was not statistically significant. It was also worth noting that while some interaction pairs displayed a trend toward mutual exclusivity, none of them reached statistical significance, presumably due to restricted cohort size.

### Mutational signature profiling of PSCC patients

**Figure 3:**
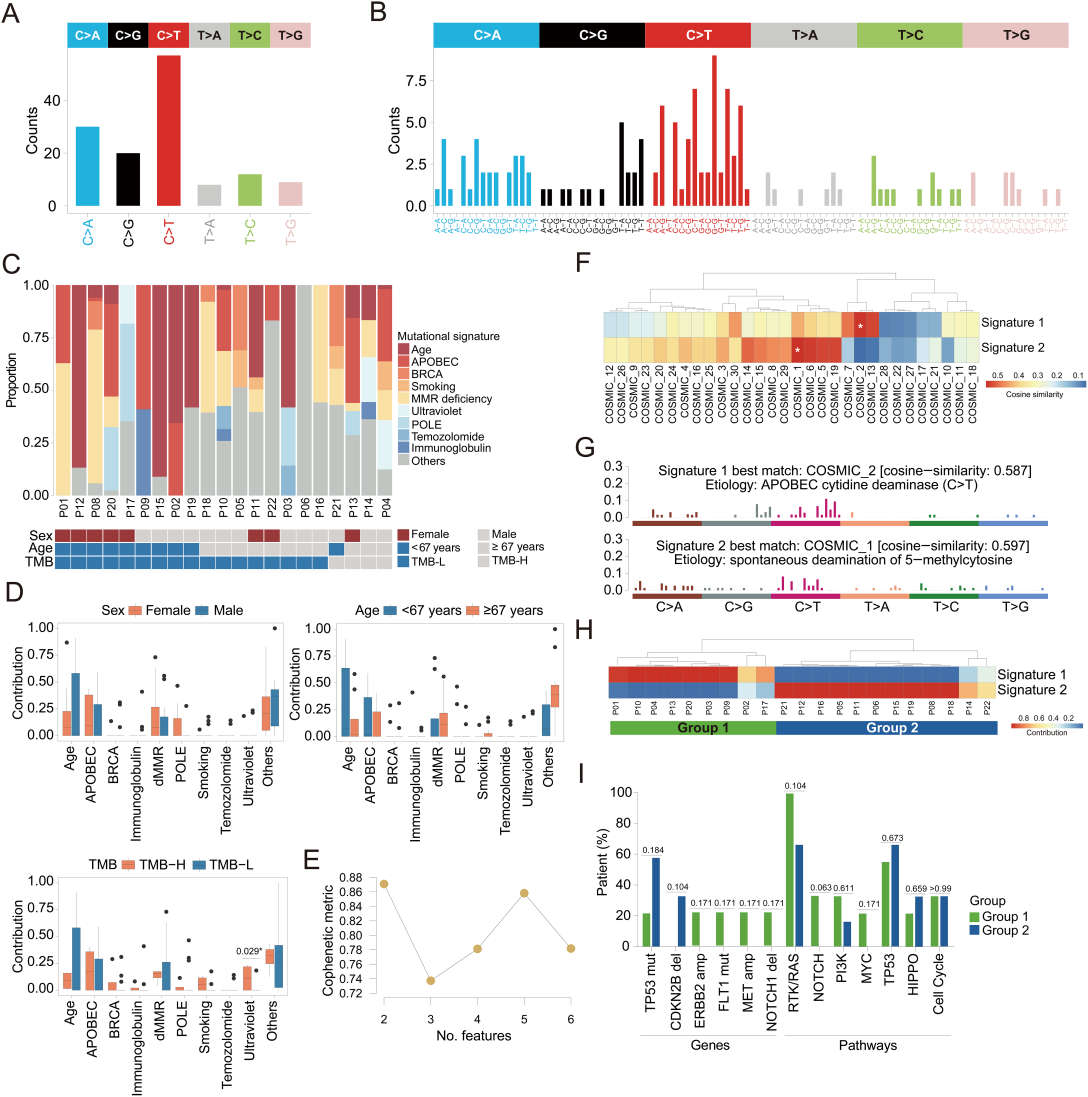
Mutational signature profiling of PSCC patients. (A) The bar plot shows the occurrence of six categories of SBS in baseline PSCC tumors (*N* = 21). (B) Signatures are displayed according to the three‐nucleotide scheme (96 mutation type classification) considering the six substitution classes (C>A, C>G, C>T, T>A, T>C, T>G), as well as 5′ and 3′ flanking bases (T, C, G, A). The *x*‐axis shows mutation types, and the *y*‐axis shows the estimated mutation counts of each mutation type. (C) The stacked bar plot shows the proportion of signatures for each patient based on the 10‐group classification. (D) Bar plots illustrate the distribution of mutational signatures in subgroups of patients stratified by clinical characteristics, including sex, age, and TMB. (E) Determination of the optimal number of mutational features based on the cophenetic metric. (F) The heatmap shows the cosine similarity of newly defined signatures 1 and 2 against validated signatures. (G) Illustration of the best matching mutational signatures (COSMIC_1 and COSMIC_2) determined by cosine similarity score. Bar plots show the relative frequencies of the 96 possible substitutions displayed in different colors below the plot. Each vertical bar represents the percentage of mutations attributed to a specific base substitution within a specific sequence context. (H) The heatmap shows the grouping of PSCC patients based on the expression patterns of the newly defined mutational signatures. (I) The bar plot shows the proportion of patients with specific gene or pathway alterations within the two patient subgroups based on mutational signature profiling.

Next, we performed an integrated mutational signature analysis using PSCC tumors from 21 patients, excluding patient 7 (P07) due to the lack of single‐base substitution (SBS) features. A total of 136 SBS were identified, with C>T (41.9%, 57/136) being the predominant mutational feature in PSCC patients, followed by C>A (22.1%, 30/136) and C>G (14.7%, 20/136) (Figure 3A). In the three‐nucleotide scheme, the most frequently mutated signatures were G[C>T]G (9/136, 6.6%), C[C>T]T (7/136, 5.1%), T[C>T]A (7/136, 5.1%), T[C>T]G (6/136, 4.4%), and A[C>T]G (6/136, 4.4%) (Figure 3B). Additionally, we also investigated the distribution of mutational signatures in patient subgroups categorized based on various clinical characteristics, including sex, age, and TMB (Figure 3C,D). Of note, the ultraviolet (UV) signature was found to be significantly associated with higher TMB in the study cohort (*p* = 0.029) (Figure 3D). Previous studies have demonstrated an interesting link between UV signature and high TMB in cutaneous primary tumors [40, 41, 42]. Hence, it is intriguing to explore whether the association between UV radiation exposure and elevated TMB varies among PSCC tumors located in different sites, such as those near pleural or hilar regions.

Using the nonnegative matrix factorization algorithm [32], we found that COSMIC_1 (aging; cosine similarity: 0.597) and COSMIC_2 (APOBEC; cosine similarity: 0.587) were the two most contributing signatures in the study cohort (Figure 3E–G). Based on the relative contributions of these two signatures, we clustered patients into two patient subgroups, with 9 patients significantly enriched with signature 1 and 12 patients predominantly enriched with signature 2 (Figure 3H). Although not reaching statistical significance, group 1 patients showed a trend toward having more genetic alterations in the RTK/RAS and PI3K pathways (*p* = 0.104 and *p* = 0.611, respectively), while those in group 2 were more likely to carry *TP53* mutations and alterations in the HIPPO pathway (*p* = 0.184 and *p* = 0.659, respectively) (Figure 3I). Despite much interest in further exploring the association between the newly defined signatures and patient prognosis, our study was hindered by the lack of patient survival data. Therefore, the functional significance of this classification for patient prognosis necessitates further investigation.

### Somatic variant detection between tumor and plasma samples

**Figure 4:**
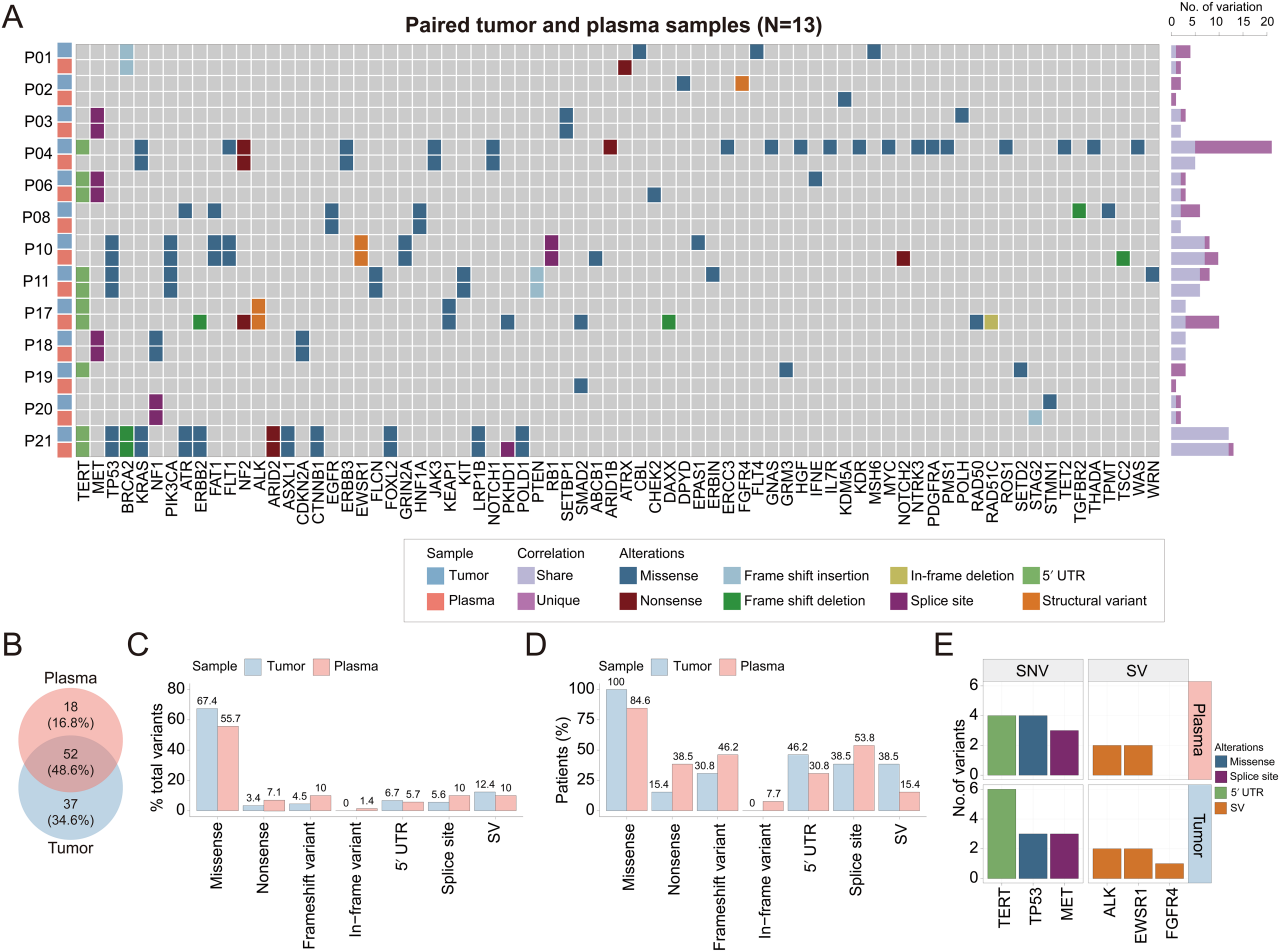
Somatic variant detection in tumor and plasma samples. (A) The heatmap shows the genomic profile for each patient in their paired tumor and plasma samples at baseline (*N* = 13). (B) The Venn diagram shows the number and percentage of shared and unique genetic alterations (excluding copy number variants) found in plasma and matched tumor tissues. (C) The bar plot shows the percentage of total variants for each somatic variation type between plasma and tumor samples. (D) The bar plot shows the proportion of patients carrying each variant type in plasma or tumor samples. (E) The bar plot shows the number of variants for specific genes in PSCC patients, grouped by variant type.

While primary tumor sequencing provides a comprehensive view of the tumor genome, it is important to acknowledge that obtaining access to primary tumors may not always be feasible. In this regard, plasma‐circulating tumor DNA (ctDNA), which is released from tumor cells into the circulation, may fulfill the role of primary tumor sampling for somatic variant detection. However, due to high levels of biases and artifacts from small fractions of ctDNA within whole blood circulating cfDNA, CNV calling from liquid biopsy‐based ctDNA profiling is challenging [43]. Therefore, in our study, we focused on the detection of somatic variants, excluding CNVs, between matched tumor and plasma samples. Here, a total of 13 PSCC patients, whose tumor and/or plasma samples had genetic alterations other than CNVs, were eligible for further analysis (Figure 4A). Of all genetic variants identified in both sample types, 52 (48.6%) alterations were mutually found, while 37 (34.6%) and 18 (16.8%) alterations were exclusively identified in tumor and plasma samples, respectively (Figure 4B). Notably, plasma samples demonstrated a similar detection rate for all types of genetic variants under examination, with differences that were not statistically significant (Figure 4C,D). Moreover, among genes with a minimum of three variant counts in both sample types, plasma samples successfully identified all three oncogenic *METex14* alterations that were also observed in paired tumor samples (Figure 4E). Furthermore, known oncogenic drivers, including *EGFR* L858R, *KRAS* mutations, and gene rearrangements in *ALK* and *EWSR1*, were detected in both sample types (Figure 4A,E).

Collectively, we showed that plasma ctDNA was efficient in identifying tumor‐derived DNA abnormalities that might be used as a baseline for informing treatment decision‐making for this poorly characterized malignancy, particularly in cases when access to the patient's tumor samples may be limited or unavailable.

### Representative patient cases propose promising treatment modalities for PSCC

**Figure 5:**
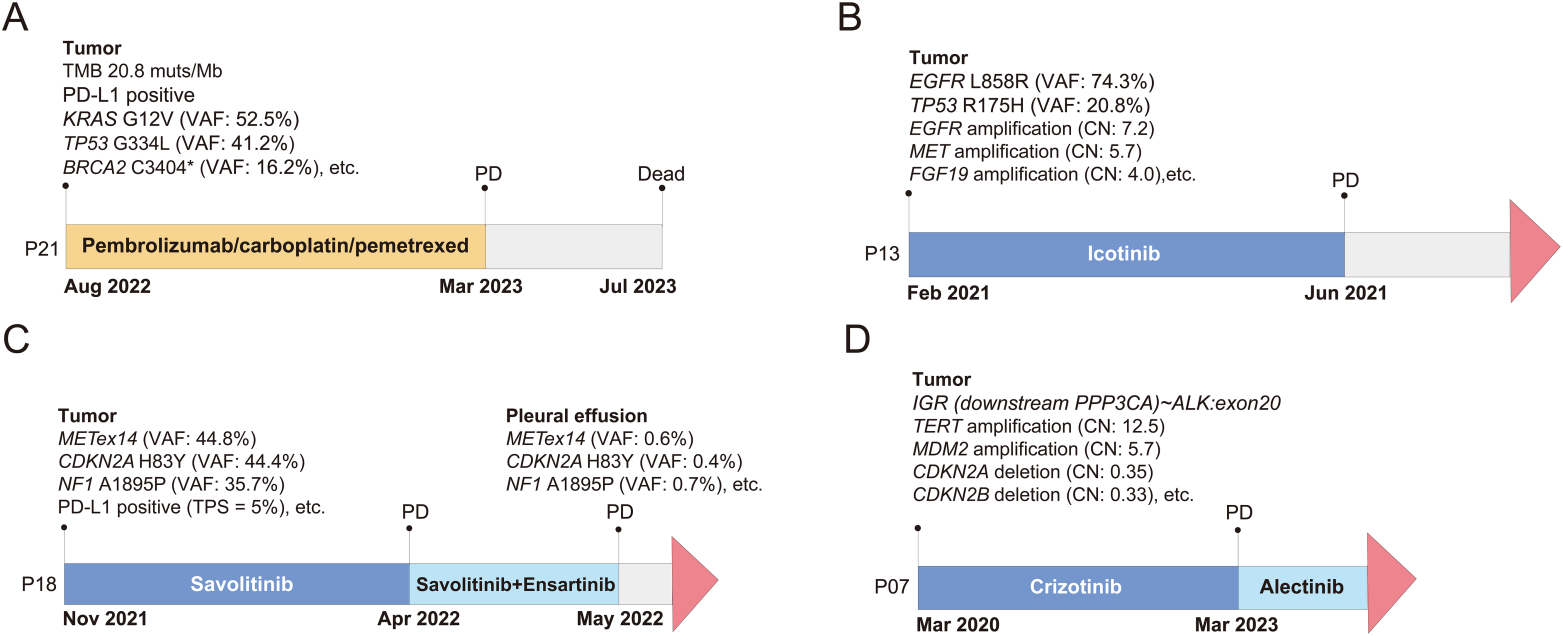
Representative patient cases. (A) Patient 21 (P21), who presents high tumor mutation load and positive PD‐L1 expression (see supplementary material, Figure S4), showed no PD for 7 months after frontline treatment with immunotherapy plus chemotherapy combination therapy. (B) Patient 13 (P13) developed PD after 4 months of treatment with icotinib, a first‐generation EGFR‐TKI. (C) Savolitinib, a selective MET tyrosine‐kinase inhibitor, was administered to patient 18 (P18) for the presence of *MET* exon 14 skipping alterations (*METex14*) in the baseline tumor. However, the patient exhibited PD after 5 months of treatment. (D) Patient 7 (P07), who exhibited a baseline *IGR*–*ALK* fusion at the DNA level and a canonical *EML4*–*ALK* fusion, underwent a three‐year course of treatment with crizotinib until the disease progressed.

Because of the rarity of PSCC, a standard treatment regimen for individuals diagnosed with this malignancy is currently lacking. Indeed, the existing literature consists of only a few case series that have reported on the clinical outcomes of PSCC patients who underwent various treatment approaches, including tyrosine kinase inhibitors (TKIs), immunotherapy, and even traditional Chinese medicine (supplementary material, Table S2) [13, 15, 44]. In this study, we discuss four patients to explore the survival outcomes of PSCC patients who received different therapeutic modalities (supplementary material, Figure S1). Patient 21 (P21), who exhibited high TMB (20.8 muts/Mb) and positive PD‐L1 expression (TPS = 30%), did not show signs of progressive disease (PD) until 7 months later (Figure 5A). On the other hand, patients 13 and 18 received icotinib and savolitinib, due to the presence of *EGFR* L858R and *METex14* in baseline tumors, respectively (Figure 5B,C). Despite initial response to therapy, both patients reached PD within 6 months, which was notably shorter than the median progression‐free survival (PFS) previously reported for these two TKIs (11.2 months for icotinib and 6.9 months for savolitinib) [45, 46]. In marked contrast, P07 who was found to be positive for a non‐canonical *ALK* fusion at the DNA level and a canonical *EML4*–*ALK* fusion at the RNA level, demonstrated a sustained survival benefit of over 3 years without disease progression after initial treatment with crizotinib (Table 2 and Figure 5D).

Moreover, we compared the molecular characteristics of PSCC and PC patients using an external cohort (supplementary material, Table S3). Despite a notable difference in the proportion of patients with *MET* variants (44.4% versus 6.8%, *p* = 0.012), the overall mutational profiles of the two histological subtypes appeared to be similar (supplementary material, Figure S3A,B). However, PSCC patients had shorter overall survival compared to those with PC (median: 11.9 versus 24.5 months, *p* = 0.35) (supplementary material, Figure S3C). Whether the varying prognoses between these two histological subtypes represent underlying biological differences or analytic variation caused by the small sample size remains to be determined.

### Germline mutation profiling for PSCC diagnosis and risk assessment

The screening for hereditary mutations has enabled better management of extracutaneous cancer risks. Here, we analyzed the sequencing data and compared genetic variants with previously annotated pathological variants according to the American College of Medical Genetics and Genomics (ACMG) classification. As shown in supplementary material, Table S4, germline alterations were identified in 3 of 22 patients (13.6%). Of these, two had likely pathogenic *TP53* mutations (c.548C>G and c.869_870del), and one had a pathogenic RecQ‐like helicase 4 (*RECQL4*) mutation (c.2464‐1G>A). Notably, patient 21 had no family history of cancer but was found to harbor a pathogenic mutation in *RECQL4*, which has been implicated in type II Rothmund–Thomson syndrome characterized by a premature aging phenotype [47]. Overall, our findings suggest that molecular testing for genetic predispositions offers significant clinical value for prevention, screening, and optimizing treatment for PSCC patients.

## Discussion

PSCC is a highly malignant type of NSCLC associated with exceedingly poor prognosis and limited treatment options. The development of diagnostic and therapeutic strategies for PSCC has been hampered by the lack of molecular characterization and poor understanding of underlying genomic drivers. Herein, we present results of comprehensive genomic profiling of the largest series to date of 22 PSCC patients. Our study has invaluable clinical significance for the diagnosis and management of this historically poorly characterized and difficult‐to‐treat disease.

Sequencing data of baseline tumors from 22 PSCC patients was first analyzed. *TP53*, *TERT*, *MET*, *CDKN2A/2B*, *RB1*, *LRP1B*, and *FAT1* were among the most frequently mutated genes (>15% of cases) in PSCC. Known oncogenic drivers, including *METex14*, *MET* amplifications, *EGFR* L858R, and *KRAS* G12C/V, were also identified at baseline. Indeed, these results aligned with previous studies on molecular profiling of PSC, in which *TP53*, *CDKN2A/2B*, *KRAS*, *TERT*, *MET*, and *LRP1B* were the most frequently altered genes with a mutational frequency above 10% [48]. The shared genetic changes between PSCC and PSC patients suggest that there might be common mechanisms driving the development of sarcomatoid carcinoma.

Meanwhile, we also noticed significant differences in the genomic landscape of PSCC patients compared to PSC and non‐PSC NSCLC. Prior studies have reported rare *ALK* rearrangement events in PSC patients, but heterogenous expression of *ALK* fusions in 2–7% of lung adenocarcinoma with multiple potential breakpoints in *EML4*, and alternate canonical or non‐canonical fusion partners involving *KIF5B*, *ASXL2*, *ATP6V1B1*, *PRKAR1A*, and *SPDYA* [49, 50]. Interestingly, while we detected a non‐canonical IGR–*ALK* fusion in P07 at the DNA level, a mature *EML4*–*ALK* fusion was detected using RNA‐seq. This result was consistent with Kang *et al* in that non‐canonical *ALK* fusions detected at the DNA level could result in a canonical *EML4*–*ALK* fusion mRNA [51]. Additionally, our findings emphasized the clinical significance of RNA‐based analysis, which provides solutions to several challenging issues encountered in detecting fusion events at the DNA level, such as probe coverage over lengthy intronic regions containing repetitive sequences, high GC content, and the inability to detect complex genomic events [52]. As there is currently no perfect assay capable of identifying 100% of actionable alterations in patient samples, multiple molecular testing methodologies are recommended, for example, integrating targeted NGS with RNA panel sequencing to gain a more comprehensive understanding of potential SVs associated with PSCC. Additionally, DNA repair‐associated genes, such as *BRCA1* and *BRCA2*, were not enriched in PSC [49]; however, they were identified in PSCC patients at mutational frequencies of 4.5% and 13.6%, respectively. Moreover, although *TP53* was the most frequently mutated gene in PSCC, the frequency of *TP53* in our study cohort was similar to that in the lung adenocarcinoma data set (46%) but significantly lower than the frequency in PSC patients (74%) [49, 53]. Collectively, the genomic landscape of PSCC patients showed significant differences when compared to other NSCLCs, with the presence of *ALK* fusions, DNA repair‐associated gene mutations, and a distinct *TP53* mutation frequency.

Importantly, we identified potentially actionable targets recommended for testing in 81.8% of PSCC patients, suggesting that patients with this rare malignancy may likely benefit from precision‐based targeted therapy. Prior studies have reported a 0–30% frequency of *BRAF* mutation in PSC, and vemurafenib treatment conferring an excellent and durable response to therapy in *BRAF* V600E‐mutated PSC patients [48, 53, 54, 55]. However, neither *BRAF* mutations nor *RET* rearrangements, which were previously reported in PSC, could be identified in PSCC. On the other hand, frequencies of activating mutations in *MET* (*METex14* and *MET* amplification), *ERBB2*, *ROS1*, and *ALK*, were significantly higher compared to PSC and non‐squamous NSCLC [48, 56], delineating possible genomic features of PSCC to the matching targeted therapies. However, according to our case study, the efficacy of icotinib and savolitinib for *EGFR* L858R‐mutated and *METex14*‐positive PSCC patients fell short of our expectations. In contrast, crizotinib exhibited a remarkable 3‐year PFS for a patient with an *ALK* rearrangement. Nevertheless, further investigation is warranted to reach a more definitive conclusion regarding the association between actionable targets and patient prognosis.

In addition to targeted therapy, other treatment approaches may also be considered for PSCC patients, which might be determined depending on cancer staging and molecular biomarkers. TMB, as a surrogate for neoantigen load, possibly reflects the ability to generate immunogenic peptides and thereby influence ICI response in patients [57]. The median TMB of our series of PSCC is 5.5 muts/Mb, which was lower than in PSC (median: 8.1 muts/Mb) and in other NSCLCs (median: 7.2 muts/Mb), but slightly higher than other solid tumors (median: 3.8 muts/Mb). In our study, TMB‐H likely co‐occurred with *KRAS* G12C, *FOXL2*, *ARID2*, and *LRP1B* mutations, which may justify the use of combination therapy involving ICIs and targeted therapies. On the other hand, although PD‐L1 expression was the first biomarker developed for clinical benefit of ICI treatment, its expression in tissue is highly variable and the predictive role of PD‐L1 might be affected by tumor histology. Interestingly, our patient case study indicates that chemoimmunotherapy might be a promising treatment option for PSCC patients with a high TMB and positive PD‐L1 expression. Nevertheless, accumulation of additional case studies and results from clinical trials is warranted to provide important treatment options for PSCC.

To our knowledge, we are presenting the largest cohort to date of patients diagnosed with PSCC given the rarity of the disease. However, this study exhibits several limitations that require consideration. First, it is crucial to recognize that our study included patients with advanced disease, for whom only biopsy samples were accessible for analysis. This could be attributed in part to the aggressive nature of PSCC, leading to diagnoses mostly at late stages. Although a panel of IHC markers along with conventional morphological assessment were carefully employed for histologic typing, a cautious approach to these results is recommended. Second, this retrospective analysis was limited by the absence of detailed clinical information for patients, which may impede the comprehensive analysis of this rare malignancy. Third, while our mutational signature analysis facilitates patient stratification, the clinical significance of grouping patients remains largely unknown due to limited sample size and a dearth of survival data. Therefore, it is imperative to utilize larger cohorts and conduct more comprehensive analyses in future studies to assess the prognostic value of the two newly defined mutational signatures in our study.

In conclusion, we provide a comprehensive genomic characterization of PSCC, which may offer valuable insights for the identification of therapeutic targets and the development of effective therapeutic strategies for this poorly characterized cancer. Our findings also highlight the clinical significance of plasma ctDNA in identifying tumor‐derived genomic abnormalities, which might be exploited for molecular characterization and inform treatment decisions.

## Author contributions statement

YS and SQ designed the study. YS and SQ performed the data acquisition. YS, SQ, SW and JP performed data analysis. YS, SQ, SW and QO edited the manuscript. WL and HZ supervised the present study. All authors read and approved the final manuscript.

## Supporting information


**Figure S1.** Flowchart of the study design
**Figure S2.** Tumor‐derived genomic features in PSCC patients
**Figure S3.** Genomic and prognosis analysis using the external dataset
**Figure S4.** Quantification of PD‐L1 expression in the tumor sample from patient 21
**Table S1.** List of the 201 genes covered by panel RNA sequencing
**Table S2.** Summary of previous findings for PSCC
**Table S3.** Clinical characteristics of patients in the external dataset
**Table S4.** Pathogenic and likely pathogenic germline mutations in PSCC patients

## Data Availability

The datasets generated and/or analyzed during this current study are available from the corresponding author upon reasonable request.
