# Supplementary material for: Comprehensive genomic profiling of pulmonary spindle cell carcinoma using tissue and plasma samples: insights from a real‐world cohort analysis
Source: J Pathol Clin Res. 2024 Apr 25;10(3):e12375. doi: 10.1002/2056-4538.12375 (PMC11044156; doi:10.1002/2056-4538.12375)
Supplement: Supplementary file 1 — Figure S1. Flowchart of the study design Figure S2. Tumor‐derived genomic features in PSCC patients Figure S3. Genomic and prognosis analysis using the external dataset Figure S4. Quantification of PD‐L1 expression in the tumor sample from patient 21 Table S1. List of the 201 genes covered by panel RNA sequencing Table S2. Summary of previous findings for PSCC Table S3. Clinical characteristics of patients in the external dataset Table S4. Pathogenic and likely pathogenic germline mutations in PSCC patients [file CJP2-10-e12375-s001.pdf]

Comprehensive genomic profiling of pulmonary spindle cell carcinoma using tissue and plasma samples: insights from a real-world cohort analysis

Y Sun, S Qin *et al.*, *J Pathol Clin Res*, https://doi.org/10.1002/2056-4538.12375

Supplementary Figures S1–S4
Supplementary Tables S1–S4

References numbers refer to the list in the main paper

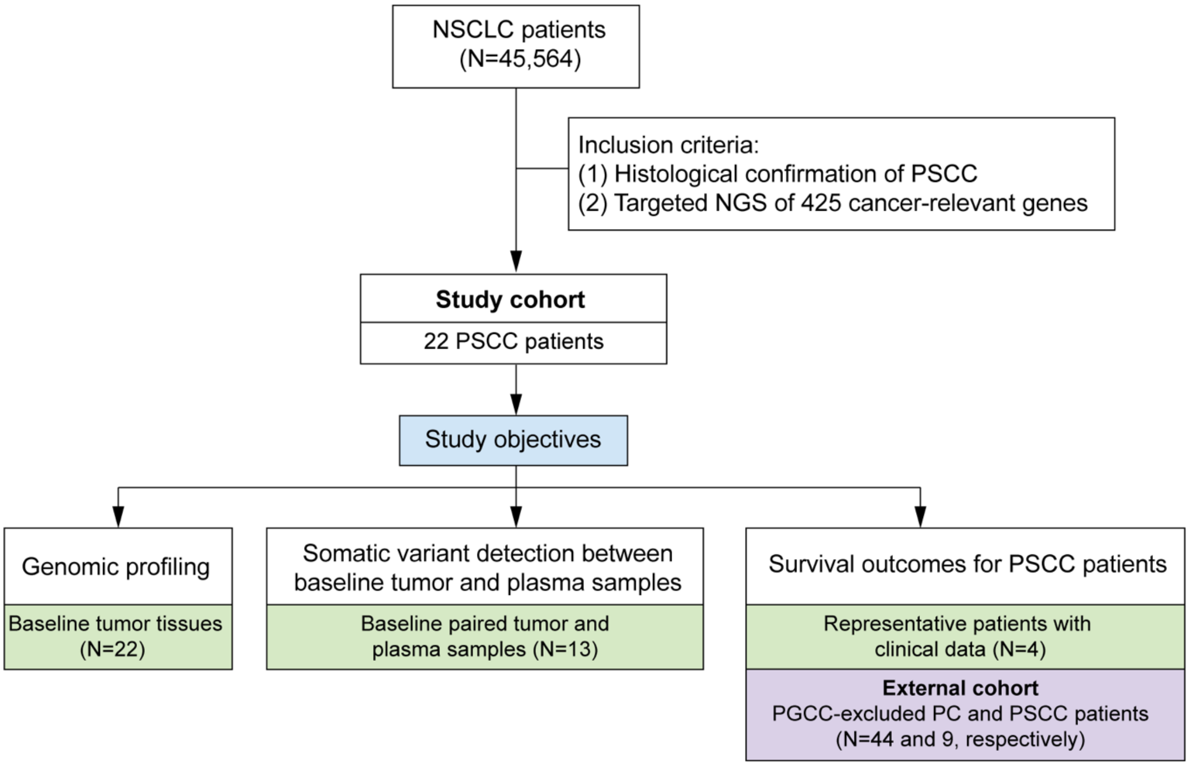

Figure S1. Flowchart of the study design.
The study cohort included twenty-two patients histologically diagnosed with pulmonary spindle cell carcinoma (PSCC) from an extensive database search of over 45,564 NSCLC patients. Baseline tumor samples from all 22 patients were analyzed by targeted next-generation sequencing (NGS) to delineate the mutational landscape of PSCC. Additionally, plasma samples from 13 of these patients were analyzed to compare somatic variant detection compared to that of baseline tumors. Four patients in the study cohort with complete clinical data were utilized to explore the therapeutic implications of chemotherapy/immunotherapy and tyrosine kinase inhibitors in PSCC patients. An external cohort consisting of 9 PSCC and 44 PC patients was employed to study genomic differences between the two histological types and patient prognosis.

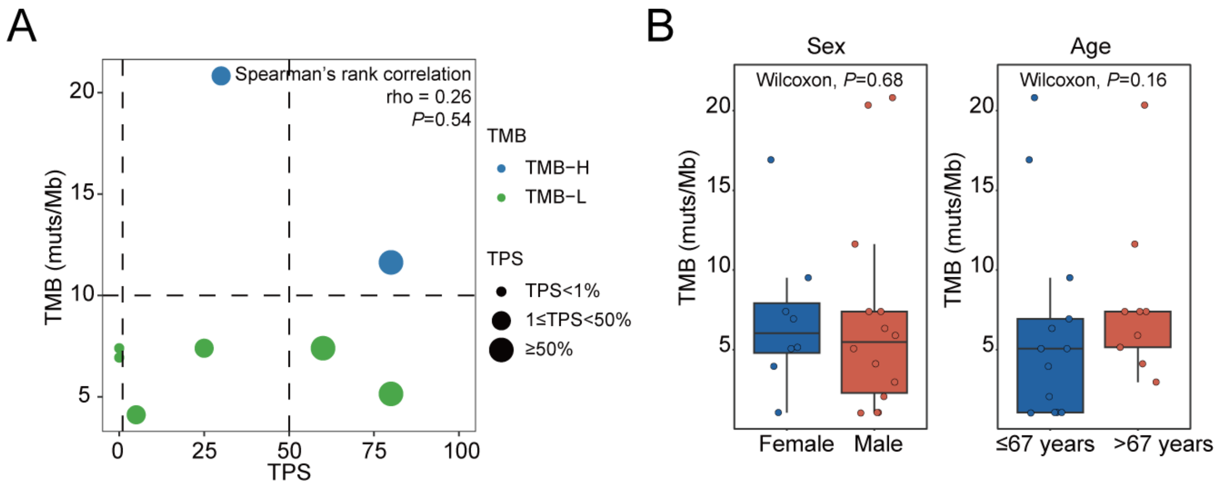

Figure S2. Tumor-derived genomic features in PSCC patients.
(A) The correlation between tumor mutation burden (TMB) and tumor proportion score (TPS) of PSCC patients in the study cohort (N=8). Dot size represents TPS indicative of PD-L1 expression in baseline tumors. Blue denotes TMB-H, whereas green denotes TMB-L tumors. **(B)** Box plots demonstrate the distribution of TMB in patients stratified by sex or age.

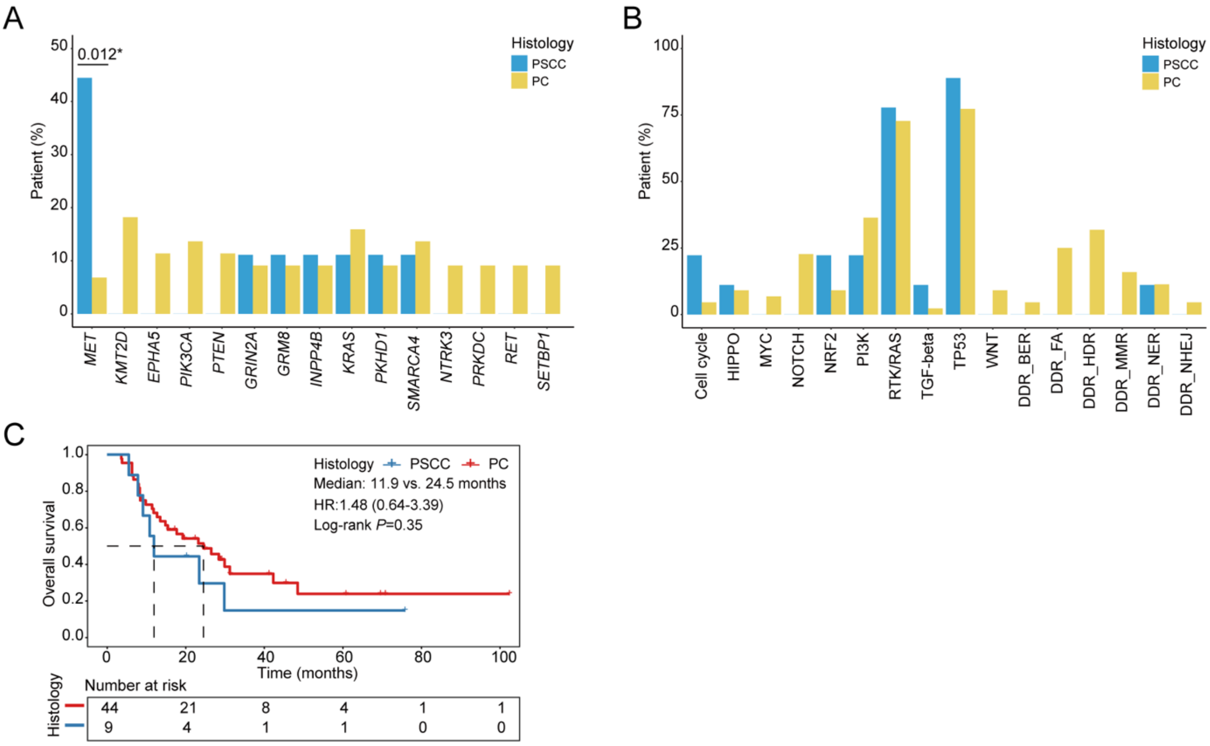

Figure S3. Genomic and prognosis analysis using the external dataset.
(A, B) Bar plots showing the proportion of patients harboring selective genes (A) and pathway alterations (B) in subgroup patients in strata of histology. **(C)** Kaplan-Meier curves demonstrate the overall survival of PSCC compared to PC patients.

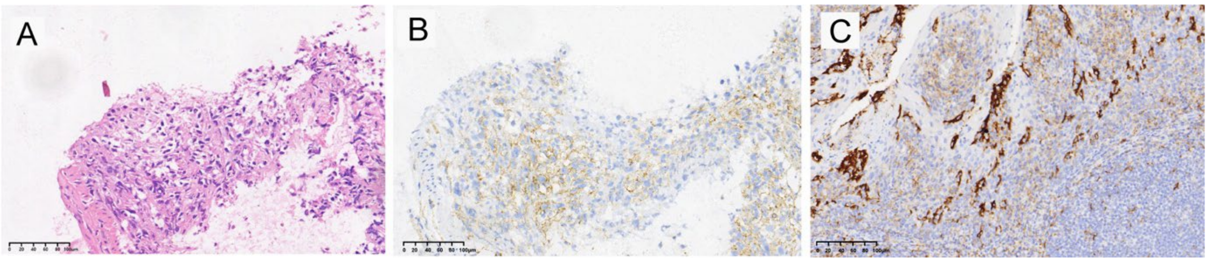

Figure S4. Quantification of PD-L1 expression in the tumor sample from patient 21.
(**A**) Morphological characteristics of pulmonary spindle cell carcinoma evaluated using hematoxylin and eosin staining. (**B**) Immunohistochemical analysis revealed positive membranous PD-L1 staining in the tumor sample. (**C**) The strong staining reaction observed in the reticulated epithelium of the deep tonsillar crypts was used as the positive control, whereas the absence of staining on the surface epithelium of tonsils served as the negative control.

Table S1. List of the 201 genes covered by panel RNA sequencing

ACLY	*ACTB*	*ACTG2*	*ACVR2A*	*ADAP2*	*AHRR*
ALK	*ASPSCR1*	*ARHGEF2*	*ATF1*	*BAG4*	*BCLAF1*
BCOR	*BCORL1*	*BRAF*	*BRD8*	*BTBD9*	*C11orf95*
CAMTA1	*CAND1*	*CARS1*	*CCDC157*	*CCDC170*	*CCNB3*
CD74	*CDH11*	*CDH17*	*CDH18*	*CIC*	*CITED2*
CLTC	*COL1A1*	*COL6A3*	*COP1*	*CREB1*	*CREB3L1*
CREB3L2	*CREBBP*	*CSF1*	*CTDP1*	*CTNNB1*	*CYBRD1*
DDIT3	*DNAH17*	*DNMT1*	*E2F1*	*EML4*	*EP400*
EPB41L2	*EPC1*	*EPC2*	*ERG*	*ESR1*	*ETV1*
ETV4	*ETV6*	*EWSR1*	*FEV*	*FGFR1*	*FGFR2*
FGFR3	*FLI1*	*FN1*	*FOSB*	*FOXO1*	*FOXO4*
FUS	*FYN*	*GCGR*	*GLI1*	*GLIS3*	*GLT8D2*
GREB1	*GRM1*	*HECTD4*	*HEY1*	*HMGA2*	*IGF2*
IGFL2	*IRX2*	*JAZF1*	*KDM2A*	*KDM6A*	*KLF17*
KMT2A	*LMNA*	*LRP1*	*MALAT1*	*MAML2*	*MAML3*
MAMLD1	*MBTD1*	*MEAF6*	*MED12*	*MEF2A*	*MEIS1*
MET	*MTHFD1*	*MYB*	*MYBL1*	*MYH9*	*MYLK*
NAB2	*NCOA1*	*NCOA2*	*NCOR2*	*NDRG1*	*NFATC1*
NFATC2	*NFIB*	*NOC4L*	*NONO*	*NPEPPS*	*NR4A3*
NRG1	*NTRK1*	*NTRK2*	*NTRK3*	*NUTM1*	*NUP210L*
NUTM2A	*PATZ1*	*PAX3*	*PAX7*	*PBX1*	*PBX3*
PDGFB	*PDGFD*	*PHF1*	*PLAG1*	*POU5F1*	*PRDM10*
PRRX1	*PTCH1*	*RAD51B*	*RAF1*	*RANBP2*	*RBFOX2*
RBPMS	*RELA*	*RET*	*RNF130*	*ROS1*	*SDC4*
SERPINE1	*SFPQ*	*SLC39A5*	*SLC3A2*	*SMARCA2*	*SMARCA5*
SND1	*SNRNP25*	*SP3*	*SRF*	*SS18*	*SS18L1*
SSX1	*SSX2*	*SSX4*	*STAT6*	*SUZ12*	*TACC1*
TAF15	*TAFA2*	*TCF12*	*TEAD1*	*TERT*	*TESPA1*
TFCP2	*TFE3*	*TFG*	*THBS1*	*TMPRSS2*	*TNC*
TPM3	*TPM4*	*TRIO*	*TRPS1*	*TTC28*	*TTYH3*
UCK2	*USP6*	*VCAM1*	*VCP*	*VGLL2*	*VMP1*
WT1	*WWTR1*	*YAP1*	*YWHAE*	*YY1*	*ZC3H7B*
ZNF384	*ZNF444*	*ZNF521*	*ADGRF5*	*C5orf38*	*DUX4*
FOS	*NUTM2B*	*ZFP36*			

Table S2. Summary of previous findings for PSCC

Reference	Cohort	PSCC	Key research findings	Treatment response
Fallet *et al* [54]	81	4	*KRAS*, *EGFR*, *TP53*, *STK11*, *NOTCH1*, *NRAS*, and *PI3KCA* mutations were frequently identified in PSC.	~
Terra *et al* [58]	34	8	PSC tumors bear known actionable targets, including *BRAF*, *NRAS*, *PIK3CA*, *AKT1*, and *ALK* fusion.	~
Saffroy *et al* [59]	81	4	PSC tumors harbor oncogenic drivers, including *KRAS*, *EGFR*, *PI3KCA*, and *BRAF*, as well as four *MET* exon 14 mutations.	~
Ikushima *et al* [44] [a]	1	1	Gefitinib as first-line therapy for *EGFR* exon 19 deletion patients diagnosed with PSCC	No significant effect
Li *et al* [13] [a]	1	1	Long-term survival was observed in a patient with PSCC who underwent treatment with traditional Chinese medicine.	A 48-month survival after diagnosis
Tsurumi *et al* [15] [a]	1	1	Pembrolizumab was administered due to the high expression of PD-L1 in more than 90% of tumor cells.	The tumor decreased in size after 9 weeks;<br>Tumor regression was maintained after 21 weeks
Zhang *et al* [60]	38	22	PSC patients harbor different genetic and immunologic features. *SARS* mutations were associated with worse overall survival in PSC patients	~

[a]Case report

Abbreviations: PSC, pulmonary sarcomatoid carcinoma; PSCC, pulmonary spindle cell carcinoma

Table S3. Clinical characteristics of patients in the external dataset (N=53)

Characteristic	PSCC (N=9)	PC (N=44)	*p* value*
Age at diagnosis (years)			>0.99
<63	4 (44.4%)	22 (50.0%)	
⩾63	5 (55.6%)	22 (50.0%)	
Sex			0.09
Female	0 (0.00%)	14 (31.8%)	
Male	9 (100%)	30 (68.2%)	
Clinical stage			0.64
I	0 (0.00%)	7 (15.9%)	
II	3 (33.3%)	12 (27.3%)	
III	5 (55.6%)	22 (50.0%)	
IV	1 (11.1%)	3 (6.82%)	
Smoking history			0.25
Non-smoker	1 (11.1%)	15 (34.1%)	
Smoker	8 (88.9%)	29 (65.9%)	
Family history of cancer			>0.99
No	7 (77.8%)	31 (70.5%)	
Yes	2 (22.2%)	13 (29.5%)	
Lymph node metastasis			0.49
No	6 (66.7%)	23 (52.3%)	
Yes	3 (33.3%)	21 (47.7%)	
PD-L1 expression			>0.99
Negative	5 (55.6%)	19 (43.2%)	
Positive	4 (44.4%)	14 (31.8%)	
Unknown	0 (0.00%)	11 (25.0%)	

**p* values were calculated based on Fisher's exact test.

Table S4. Pathogenic and likely pathogenic germline mutations in PSCC patients

ID	Age	Sex	Family history of cancer	Gene	AA Change	Mutation type	ACMG classification
P05	79	Male	Liver cancer; Gastric cancer	*TP53*	c.548C>G (p.S183*)	Nonsense mutation	Likely pathogenic
P20	52	Female	Cervical cancer	*TP53*	c.869_870del (p. R290Qfs*15)	Frameshift deletion	Likely pathogenic
P21	62	Male	None	*RECQL4*	c.2464-1G>A	Intron variant	Pathogenic
